# Androgen-deprivation therapy and the risk of newly developed fractures in patients with prostate cancer: a nationwide cohort study in Korea

**DOI:** 10.1038/s41598-021-89589-3

**Published:** 2021-05-12

**Authors:** Do Kyung Kim, Hye Sun Lee, Ju-Young Park, Jong Won Kim, Hyun Kyu Ahn, Jee Soo Ha, Kang Su Cho

**Affiliations:** 1 Department of Urology, Soonchunhyang University Hospital, Soonchunhyang University College of Medicine, Seoul, Republic of Korea; 2 Biostatistics Collaboration Unit, Yonsei University College of Medicine, Seoul, Republic of Korea; 3 Department of Statistics and Data Science, Yonsei University, Seoul, Republic of Korea; 4 Department of Urology, Inha University School of Medicine, Seoul, Republic of Korea; 5 Department of Urology, Ewha Womans University Medical Center, Seoul, Republic of Korea; 6 Department of Urology, Prostate Cancer Center, Gangnam Severance Hospital, Urological Science Institute, Yonsei University College of Medicine, 211 Eonju-ro, Gangnam-gu, Seoul, 06273 Republic of Korea

**Keywords:** Cancer, Prostate cancer

## Abstract

We evaluated the risk of osteoporosis and fractures associated with androgen deprivation therapy (ADT) use and duration in men with prostate cancer. From the nationwide claims database in South Korea, a total of 218,203 men with prostate cancer were identified between 2008 and 2017. After applying the inclusion and exclusion criteria, a total of 144,670 patients were included in the analysis. To adjust for comorbidities between cohorts, 1:1 propensity score matching was used. Cox proportional hazard regression models were used to estimate adjusted hazard ratios (HRs) and 95% confidence intervals (CIs) of events associated with ADT, after controlling for potential confounding factors. In the matched cohort, there were differences in the incidence of newly developed osteoporosis (8.79% in the ADT group vs. 7.08% in the non-ADT group, p < 0.0001) and fractures (8.12% in the ADT group vs. 5.04% in the non-ADT group, p < 0.0001). Age-adjusted Cox regression analysis revealed that the ADT group had a significantly higher risk of osteoporosis (HR, 1.381; 95% CI, 1.305–1.461; p < 0.0001) and fractures (HR, 1.815; 95% CI, 1.703–1.935; p < 0.0001) compared to the non-ADT group. Furthermore, the risk of osteoporosis and fractures increased as the duration of ADT increased. The ADT was associated with an increased risk of osteoporosis and fractures in prostate cancer patients. Clinicians who administer ADT for patients with prostate cancer should always be mindful of the risk of osteoporosis and fracture, avoid unnecessary ADT, and perform regular bone health check-ups.

## Introduction

Androgen deprivation therapy (ADT) is a common therapy for men with metastatic prostate cancer; those with locally advanced disease, in combination with radical surgery or radiation therapy; and those with biochemical recurrence after definitive treatment^1^. ADT in the form of orchiectomy, gonadotropin-releasing hormone (GnRH) agonists or antagonists, and anti-androgens has a beneficial effect on the prognosis of advanced disease^2–5^. Meng et al. identified that nearly 50% of patients with prostate cancer in their study cohort received ADT at some point after diagnosis^6^.

As the use of ADT increases, potentially serious side effects, including diabetes, cardiovascular disease, osteoporosis, and fractures, are also increasing^7–9^. Long-term ADT causes significant changes in physiological bone turnover, most of which lead to skeletal-related events, including spinal cord compression and pathologic fractures^10,11^. Of the many adverse effects, osteoporosis and bone fractures are particularly important in relation to increased mortality in prostate cancer^12^. Therefore, it is important to have accurate information regarding the adverse effects of ADT^13,14^. Several Western population-based studies revealed that ADT accelerated bone loss within the first 6–12 months^15,16^, and is associated with constant loss of bone during long-term treatment^17,18^. By analyzing data from the SEER–Medicare database, Shahinian et al*.* reported that GnRH-agonist may increase the risk of fracture by 1.5-fold and the incidence of hospitalization due to fractures by 1.7-fold^7^. Abrahamsen et al. reported that ADT was associated with a high risk of fracture in Danish men with prostate cancer^19^. Alibhai et al. also reported that ADT for at least 6 months is associated with an increased risk of fracture, according to Canada's population database^20^.

One study showed racial differences in bone mineral density and fractures in men receiving ADT for prostate cancer^21^, but only a few studies have been conducted regarding the risk of fracture with ADT in the Asian population. To the best of our knowledge, only one population-based study has been conducted concerning this issue in an Asian population, in Taiwan^22^, raising the possibility that the harmful effects of ADT on the risk of fracture in the Asian population may be less than those in the Western population. In the present study, we evaluated the risk of newly developed osteoporosis and fracture following ADT in men with prostate cancer using the nationwide health insurance claims database in Korea.

## Results

### Patient characteristics

**Figure 1:**
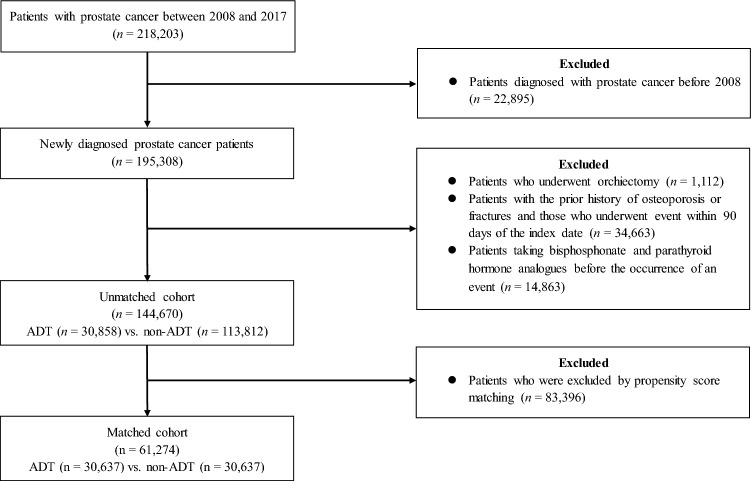
Flow diagram outlining the enrollment of the patient cohort.

A total of 144,670 men with prostate cancer fulfilled all inclusion and exclusion criteria (Fig. 1). Of these patients, 30,858 men constituted the ADT group and 113,812 men constituted the non-ADT group. After 1:1 propensity score matching, 30,637 patients from each group were selected. In the matched cohort, significant differences were not noted with respect to the baseline covariates between the two groups, except for the follow-up duration (1317.42 ± 920.72 days in the ADT group vs. 1453.65 ± 1009.91 days in the non-ADT group; p < 0.0001; Table 1).

**Table 1:** Patient demographic characteristics.

Variables	Unmatched cohort			Matched cohort		
	ADT (n = 30,858)	Non-ADT (n = 113,812)	p-value	ADT (n = 30,637)	Non-ADT (n = 30,637)	p-value
Age, year (mean ± SD)	72.28 ± 8.03	64.10 ± 10.18	< 0.0001	72.19 ± 7.98	72.19 ± 7.98	0.9879
Rheumatoid arthritis, n (%)	1614 (5.23)	6525 (5.73)	0.0007	1604 (5.24)	1582 (5.16)	0.6889
Diabetes mellitus, n (%)	11,392 (36.92)	36,941 (32.46)	< 0.0001	11,295 (36.87)	11,323 (36.96)	0.8147
Hyperthyroidism, n (%)	566 (1.83)	2808 (2.47)	< 0.0001	564 (1.84)	541 (1.77)	0.4850
Chronic liver disease, n (%)	3148 (10.20)	14,160 (12.44)	< 0.0001	3128 (10.21)	3167 (10.34)	0.6038
Chronic kidney disease, n (%)	1130 (3.66)	2450 (2.15)	< 0.0001	1111 (3.63)	1076 (3.51)	0.4460
COPD, n (%)	4408 (14.28)	11,254 (9.89)	< 0.0001	4379 (14.29)	4419 (14.42)	0.6449
Neurological disease, n (%)	5305 (17.19)	13,392 (11.77)	< 0.0001	5261 (17.17)	5303 (17.31)	0.6533
Follow-up duration, day (mean ± SD)	1314.39 ± 919.90	1594.08 ± 1035.78	< 0.0001	1317.42 ± 920.72	1453.65 ± 1009.91	< 0.0001
**Duration of ADT**						
< 1 year	10,138 (32.85)			10,060 (32.84)		
1–2 years	7875 (25.52)			7803 (25.47)		
2–3 years	5006 (16.22)			4968 (16.22)		
> 3 years	7839 (25.40)			7806 (25.48)		
Osteoporosis, n (%)	2709 (8.78)	6007 (5.28)	< 0.0001	2693 (8.79)	2168 (7.08)	< 0.0001
Fracture, n (%)	2506 (8.12)	3959 (3.48)	< 0.0001	2487 (8.12)	1544 (5.04)	< 0.0001
**The sites of fracture**						
Hip	581 (1.88)	771 (0.68)		574 (1.87)	391 (1.28)	
Spine	1299 (4.21)	2134 (1.88)		1289 (4.21)	844 (2.75)	
Upper extremity	609 (1.97)	1039 (0.91)		607 (1.98)	302 (0.99)	
Multiple sites	17 (0.06)	15 (0.01)		17 (0.06)	7 (0.02)	

*ADT* androgen deprivation therapy, *COPD* chronic obstructive pulmonary disease, *SD* standard deviation.

### Osteoporosis risk in ADT vs. non-ADT groups

**Figure 2:**
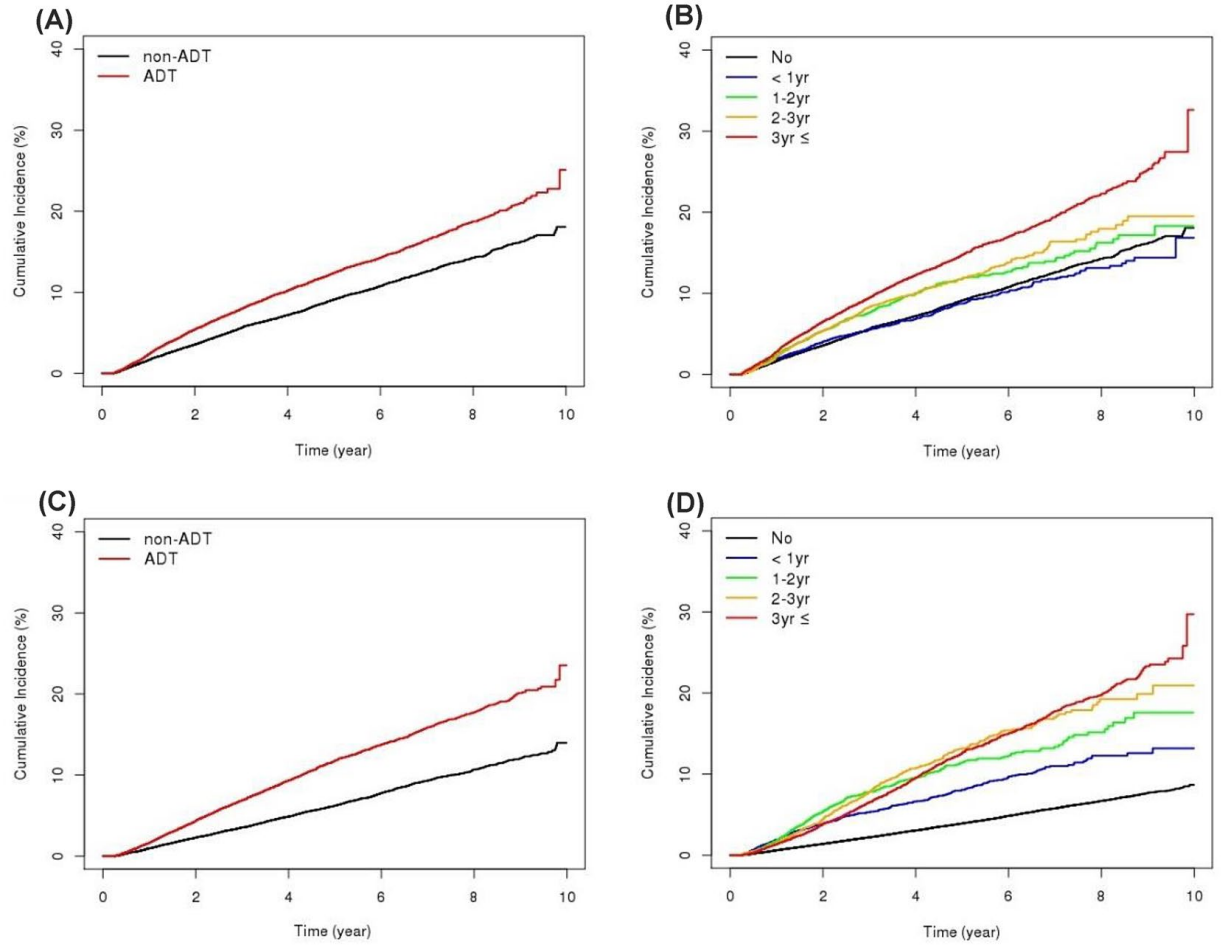
Cumulative incidences of osteoporosis and fractures in the matched cohort. (**A**) Incidence of osteoporosis according to the use of ADT. (**B**) Incidence of osteoporosis according to the duration of ADT use. (**C**) Incidence of fractures according to the use of ADT. (**D**) Fractures according to the duration of ADT use. *ADT* androgen deprivation therapy.

In the matched cohort, the ADT group had a higher proportion of newly developed osteoporosis than the non-ADT group (8.79% vs. 7.08%, respectively; p < 0.0001; Table 1). In addition, age-adjusted Cox regression analysis revealed that the ADT group had a significantly higher risk of osteoporosis compared to the non-ADT group (HR, 1.381; 95% CI, 1.305–1.461; p < 0.0001; Table 2, Fig. 2A). ADT for < 1 year did not increase the risk of osteoporosis; however, ADT for > 1 year showed an increased risk of osteoporosis compared to the non-ADT group. As the duration of ADT increased, the risk of osteoporosis also increased (HR, 1.293 for ADT administered for 1–2 years; HR, 1.352 for ADT administered for 2–3 years, and HR, 1.639 for ADT administered for > 3 years; all p < 0.001; Table 2, Fig. 2B). Even after adjusting for index date in the non-ADT group, ADT’s harmful effect in terms of osteoporosis was still apparent (Supplementary Table 3).

**Table 2:** Age-adjusted Cox regression analysis for predicting osteoporosis in the unmatched (A) and matched (B) cohorts.

Variables	Univariable analysis		Age-adjusted Cox regression analysis						
			ADT		Duration of ADT				
	HR (95% CIs)	p-value	HR (95% CIs)	p-value	HR (95% CIs)	Pairwise comparison p-value			
**(A) Unmatched cohorts**									
Age	1.065 (1.062–1.067)	< 0.0001	1.059 (1.057–1.062)	< 0.0001	1.059 (1.056–1.062)	< 0.0001			
ADT									
No	Ref.		Ref.						
Yes	2.112 (2.018–2.211)	< 0.0001	1.377 (1.312–1.445)	< 0.0001					
Duration of ADT									
No	Ref.				Ref.	Ref.			
< 1 year	1.493 (1.356–1.644)	< 0.0001			1.043 (0.947–1.150)	0.3921	Ref.		
1–2 years	2.038 (1.861–2.232)	< 0.0001			1.305 (1.190–1.432)	< 0.0001	0.0006	Ref.	
2–3 years	2.088 (1.900–2.295)	< 0.0001			1.362 (1.237–1.498)	< 0.0001	< 0.0001	0.51	Ref.
> 3 years	2.514 (2.367–2.671)	< 0.0001			1.599 (1.502–1.702)	< 0.0001	< 0.0001	0.0001	0.003
**(B) Matched cohorts**									
Age	1.036 (1.032–1.040)	< 0.0001	1.035 (1.031–1.039)	< 0.0001	1.034 (1.031–1.038)	< 0.0001			
ADT									
No	Ref.		Ref.						
Yes	1.392 (1.315–1.473)	< 0.0001	1.381 (1.305–1.461)	< 0.0001					
Duration of ADT									
No	Ref.				Ref.	Ref.			
< 1 year	0.982 (0.887–1.088)	0.7334			1.016 (0.917–1.125)	0.762	Ref.		
1–2 years	1.317 (1.195–1.452)	< 0.0001			1.293 (1.173–1.426)	< 0.0001	0.0002	Ref.	
2–3 years	1.363 (1.232–1.507)	< 0.0001			1.352 (1.222–1.495)	< 0.0001	< 0.0001	0.5174	Ref.
> 3 years	1.681 (1.568–1.801)	< 0.0001			1.639 (1.529–1.757)	< 0.0001	< 0.0001	< 0.0001	0.003

*ADT* androgen deprivation therapy, *CI* confidence interval, *HR* hazard ratio.

### Fracture risk in the ADT group vs. the non-ADT group

In the matched cohort, the ADT group had a higher proportion of fracture patients than the non-ADT group (8.12% vs. 5.04%, respectively; p < 0.0001, Table 1). In addition, there was a stronger correlation with the risk of fractures in the ADT group compared to that in the non-ADT group, as determined by the age-adjusted Cox regression analysis (HR, 1.815; 95% CI, 1.703–1.935; p < 0.0001; Table 2, Fig. 2C). The analysis of the effect of duration of ADT showed that the risk of fracture increased as the duration of ADT increased, except for the period of 1–2 years, 2–3 years, and > 3 years (Table 3, Fig. 2D). Age-adjusted Cox regression analysis for fractures after adjusting for the follow-up duration did not reveal significant differences in the results compared to those obtained before adjustment (Supplementary Table 4).

**Table 3:** Age-adjusted Cox regression analysis for predicting fractures in the unmatched (A) and matched (B) cohorts.

Variables	Univariable analysis		Age-adjusted Cox regression analysis						
			ADT		Duration of ADT				
	HR (95% CIs)	p-value	HR (95% CIs)	p-value	HR (95% CIs)	Pairwise comparison p-value			
**(A) Unmatched cohorts**									
Age	1.078 (1.075–1.081)	< 0.0001	1.066 (1.063–1.069)	< 0.0001	1.066 (1.063–1.069)	< 0.0001			
ADT									
No	Ref.		Ref.						
Yes	2.995 (2.848–3.150)	< 0.0001	1.884 (1.785–1.987)	< 0.0001					
Duration of ADT									
No	Ref.				Ref.	Ref.			
< 1 year	2.221 (2.009–2.455)	< 0.0001			1.512 (1.366–1.674)	< 0.0001	Ref.		
1–2 years	3.109 (2.830–3.417)	< 0.0001			1.915 (1.739–2.108)	< 0.0001	0.0004	Ref.	
2–3 years	3.320 (3.018–3.653)	< 0.0001			2.092 (1.898–2.306)	< 0.0001	< 0.0001	0.1688	Ref.
> 3 years	3.237 (3.028–3.460)	< 0.0001			1.978 (1.846–2.120)	< 0.0001	< 0.0001	0.5473	0.3114
**(B) Matched cohorts**									
Age	1.068 (1.064–1.073)	< 0.0001	1.068 (1.063–1.072)	< 0.0001	1.067 (1.063–1.072)	< 0.0001			
ADT									
No	Ref.		Ref.						
Yes	1.837 (1.724–1.958)	< 0.0001	1.815 (1.703–1.935)	< 0.0001					
Duration of ADT									
No	Ref.				Ref.	Ref.			
< 1 year	1.360 (1.221–1.515)	< 0.0001			1.453 (1.304–1.619)	< 0.0001	Ref.		
1–2 years	1.894 (1.709–2.098)	< 0.0001			1.833 (1.654–2.031)	< 0.0001	0.0005	Ref.	
2–3 years	2.028 (1.828–2.250)	< 0.0001			2.006 (1.808–2.225)	< 0.0001	< 0.0001	0.1635	Ref.
> 3 years	1.995 (1.847–2.155)	< 0.0001			1.911 (1.768–2.064)	< 0.0001	< 0.0001	0.4496	0.3802

*ADT* androgen deprivation therapy, *CI* confidence interval, *HR* hazard ratio.

### Risk of osteoporosis and fractures according to anti-androgen use in the ADT group

In the ADT group (n = 30,858), 90.77% of ADT users (n = 28,011) also took anti-androgens, but 9.23% of ADT users (n = 2847) did not. To validate the effect of anti-androgens on osteoporosis and fractures, the ADT group was subdivided into two groups (anti-androgen users vs. non-users) through propensity score matching according to age and duration of ADT, and 2846 patients were selected for each group. There was no difference in the risk of osteoporosis between anti-androgen users and non-users in the ADT group (HR, 1.039; 95% CI, 0.850–1.269; p = 0.712) and in the risk of fractures (HR, 1.222; 95% CI, 0.992–1.506; p = 0.0599; Supplementary Table 5).

## Discussion

In this large population-based study, we found that the use of ADT in the treatment of prostate cancer was associated with an increased risk of osteoporosis and fracture. The ADT group had a higher cumulative incidence of osteoporosis and fracture than the non-ADT group in the propensity score-matched cohort. In addition, the risk of osteoporosis and fracture increased as the duration of ADT increased, although concurrent use of anti-androgens did not increase the harmful effects of ADT.

Theories regarding osteoporotic fractures include the effect of increased bone turnover on bone strength, independent of reduced bone mineral density^23^. Normal bone is in a state of equilibrium with ongoing bone formation and resorption mediated by osteoblasts and osteoclasts, respectively^24^. Androgen deprivation induces testosterone and estrogen deficiency, increasing bone turnover and bone resorption, resulting in a decrease in the bone mineral density (BMD)^25,26^. Our previous meta-analysis showed that prostate cancer patients treated with ADT showed a significant decrease in the BMD compared to the controls^27^. Large-scale population-based studies on the correlation between ADT and fracture have been conducted in several Western countries^7,19,20,28,29^. Shahinian et al. conducted a study of 50,613 men who survived for more than 5 years after prostate cancer diagnosis, as recorded in the database of the National Cancer Institute's SEER program and Medicare^7^. The authors reported that patients using ADT experienced fractures more frequently than those not using ADT, and there was a significant relationship between fracture risk and cumulative dose of ADT in the first year. Beebe-Dimmer et al. also investigated the association between ADT and fracture risk using the SEER-Medicare dataset^28^. They reported that ADT in elderly men with prostate cancer increased the incidence of fractures, and the effect appeared to decrease with increasing time duration since the administration of the last dose of a GnRH agonist. Other population-based cohort studies conducted in Denmark, Canada, and New Zealand reported similar results.

Nevertheless, it is not known whether the effects of ADT on BMD and fractures vary among races. Morgans et al. compared BMD and fracture between African American and Caucasian men receiving ADT for prostate cancer to assess whether race-related ADT effects vary^21^. They reported that the hip BMD was higher and the prevalence of vertebral fractures was lower in African American men receiving ADT for prostate cancer than in Caucasian men. Therefore, there may be a racial difference in the effect of ADT on fractures. A few studies have discussed the fracture risk among prostate cancer patients in the Asian population receiving ADT, and it is unclear whether ADT has a similar effect on the BMD of Asian patients with prostate cancer. Several studies have suggested that there are racial differences in the bone density and incidence of fractures between Asian and Caucasian women^30–32^. Wu et al. conducted a study using the National Health Insurance Program of Taiwan^22^. They showed that ADT or orchiectomy increased the risk of fracture in Chinese patients with prostate cancer. However, the 5-year fracture-free rates were 90.0% in the ADT group and 92.2% in the non-ADT group^22^. It has been suggested that the effect of ADT on the decrease in the fracture-free rate in the Asian population may not be as marked as that reported in Western studies^7,20,33^. In our study, the 5-year fracture-free rates in the ADT group were 88.26% compared with 93.78% in the non-ADT group. Our results did not differ significantly from those of other Western studies. Therefore, it is still questionable whether the effect of ADT on fracture risk varies according to race, and additional studies are required to validate these observations.

Evidence-based management for minimizing bone loss in prostate cancer patients with ADT is important^34^. Bisphosphonates, a human monoclonal antibody (denosumab), and selective estrogen receptor modulators (e.g., raloxifene and toremifene) are available for the management of BMD loss by ADT^35^. The National Comprehensive Cancer Network Guidelines has provided much evidence for the above treatments with respect to ADT-induced bone loss^36^. Poon et al. found that all the drugs mentioned are effective in reducing the rate of bone loss in patients with non-metastatic prostate cancer using ADT, through a systematic review and network meta-analysis^35^. Moreover, clinicians should encourage lifestyle interventions and provide information about nutritional supplements to prostate cancer patients using ADT^35^. Meanwhile, clinicians administering ADT for prostate cancer patients should consider periodic BMD testing. The USA Endocrine Society and the National Comprehensive Cancer Network recommend BMD measurement in men aged 50–69 years with fracture risk factors such as use of ADT^36,37^.

Our database included almost all prostate cancer patients in Korea during the study period, resulting in a large sample size with a relatively long follow-up period. Our study design, based on strict inclusion/exclusion criteria of the study cohort and meticulous matching by various covariates, may have been helpful in reducing confounding bias. There are few studies on the effect of ADT fracture in patients with prostate cancer in the Asian population. In this respect, the present study is very timely. Contrary to our results, previous studies have reported that the effects of ADT on fractures in Asian prostate cancer patients may not be as remarkable as those reported in the Western population^22^. Further studies on the Asian population are needed to prove the difference in these results. Bone health care is one of the major issues to address when treating patients with prostate cancer. Maintaining bone health throughout prostate cancer is a prerequisite for acceptable quality of life and optimal disease course. A more detailed monitoring and treatment guideline of bone health in prostate cancer, while taking into account the independent factors of fracture risk (i.e. BMD, familiarity for fragility fractures, metabolic bone diseases, disability or high risk of fall, and age) depending on the patient's condition (non-metastatic or metastatic disease), should be established.

There were also some limitations. Claim data do not provide clinical information such as tumor stage, tumor grade, prostate specific antigen level, and BMD data. The probability of bone metastasis in the ADT group is high, and fractures related to bone metastasis could not be excluded from the analysis. However, fractures in prostate cancer due to bone metastases are known to account for only 7 to 16 percent of all fractures^38,39^. Accordingly, the increased risk of fracture from ADT would be maintained even after excluding bone metastasis-related fractures. The present study defined the index date as the start of hormone therapy in the ADT group and the time of disease diagnosis in the non-ADT group. This may lead to an immortal time bias for the non-ADT group. We applied the exclusion method to resolve immortal time bias^40^. Moreover, sensitivity analysis was performed using time-dependent Cox regression (time-varying cox regression). Time-dependent Cox regression is the most proper way to resolve immortal-time bias, since the model’s time-dependent covariate tracks whether a classifying event occurred during the estimation process^41,42^. The time-dependent Cox regression model also increases the statistical power by using the entire study follow-up data. In addition, we did not distinguish between continuous ADT use and intermittent ADT use, which may have impacted the results.

In conclusion, the use of ADT was associated with an increased risk of osteoporosis and fracture in Korean patients with prostate cancer. Moreover, our results also indicate that the risk of osteoporosis and fracture increases as the duration of ADT increases. Clinicians who administer ADT for patients with prostate cancer should always be mindful of the risk of osteoporosis and fracture, avoid unnecessary ADT, and perform regular bone health check-ups. Meanwhile, further study investigating racial differences in the effects of ADT on fracture is needed.

## Materials and methods

### Ethics and database

This study was approved by the Institutional Review Board of Yonsei University Gangnam Severance Hospital (IRB No. 3-2018-0308) and conducted in accordance with the principles of the Declaration of Helsinki. The National Health Insurance System in the Republic of Korea is a universal health coverage system that covers more than 95% of Korean residents. The Health Insurance Review and Assessment Service (HIRA) collects claim data submitted by healthcare providers for reimbursement. HIRA claims data pertains to approximately 46 million patients each year^43^.

### Inclusion and exclusion criteria

International Classification of Diseases, 10th revision (ICD-10) codes were used to identify eligible patients for the analysis. Between January 1, 2008 and December 31, 2017, a total of 218,203 men with primary malignant prostate cancer (ICD-10 code C61.0) were identified from the HIRA database. Of them, 195,308 were newly diagnosed prostate cancer patients. Patients (n = 1112) who underwent orchiectomy were excluded, because surgical castration accounted for an extremely small proportion of patients in Korea and we wanted to focus on the effect of medical castration on osteoporosis and fractures. The following exclusion criteria were also applied: (1) patients with the prior history of osteoporosis or fractures, or whose event occurred within 3 months (n = 34,663) and (2) those who had taken medications such as bisphosphonates, calcium supplements, and parathyroid hormone analogues before the events occurred (n = 14,863; Fig. 1). Finally, a total of 144,670 patients were included in the analysis.

### Definition of groups, outcomes, and covariates

The study cohort was divided into ADT and non-ADT groups. The ADT group was defined as those using at least one dose of GnRH agonist or antagonist after the diagnosis of prostate cancer. The cumulative dose of ADT was calculated as the sum of the action periods of each ADT preparation. Osteoporosis, fracture, and other comorbidities were identified thorough the ICD-10 diagnostic codes (Supplementary Table 1). Fractures were classified as hip, spine, or upper extremity, according to the site.

The incidences of osteoporosis and fracture were ascertained more than 90 days from the index date. Adjustment covariates included patient age, history of rheumatoid arthritis, diabetes, hyperthyroidism, chronic liver disease, chronic kidney disease, chronic obstructive pulmonary disease (COPD), and neurological disease (stroke, Parkinson’s disease, and dementia).

Medication history including ADT, anti-androgen, bisphosphonates, calcium supplements, and parathyroid hormone analogues were identified using the billing codes in the HIRA database (Supplementary Table 2).

### Statistical analysis

Index date was defined as the date of first ADT use in the ADT group, and as the date of prostate cancer diagnosis in the non-ADT group. We defined the end of follow-up as the date that the event occurred or the date of the last valid inpatient or outpatient record. A 1:1 exact matching was performed to balance the comorbidities between groups. For adjustment of comorbidity between cohorts, patients were matched using the following criteria: (1) age (± 0 years), (2) the comorbidities (exact match: presence and absence). Statistical comparisons of continuous variables from patient demographic information were carried out using the Student’s *t* test, and categorical variables were compared using Pearson's Chi-squared test. Cox proportional hazard regression models were used to estimate adjusted hazard ratios (HR) and 95% confidence intervals (CI) of events associated with ADT, controlling for potential confounders. Kaplan–Meier curves were generated to examine the cumulative incidence of outcomes according to ADT use and the duration of ADT. Meanwhile, when there was a difference in the definition of index date between two groups, supplementary analyses were performed after adjusting for follow-up duration, as follows. In the ADT group, median time to ADT use from the time of prostate cancer diagnosis was 24 days, thus the adjusted index date for non-ADT users was defined as the date of prostate cancer diagnosis plus the median time to ADT use in our study^44^. A p-value < 0.05 was considered statistically significant, and all statistical tests were 2-sided. All study analyses were performed using SAS System for Windows, version 9.4.

## Supplementary Information

Supplementary Tables.
