# Supplementary material for: Androgen-deprivation therapy and the risk of newly developed fractures in patients with prostate cancer: a nationwide cohort study in Korea
Source: Sci Rep. 2021 May 12;11:10057. doi: 10.1038/s41598-021-89589-3 (PMC8115250; doi:10.1038/s41598-021-89589-3)
Supplement: Supplementary file 1 — Supplementary Tables. [file 41598_2021_89589_MOESM1_ESM.docx]

**Androgen-deprivation therapy and the risk of newly developed fractures in patients with prostate cancer: a nationwide cohort study in Korea**

Do Kyung Kim^1^, Hye Sun Lee^2^, Ju-Young Park^3^, Jong Won Kim^4^, Hyun Kyu Ahn^5^, Jee Soo Ha^6^, Kang Su Cho^6^*

**Affiliations**

^1^Department of Urology, Soonchunhyang University Hospital, Soonchunhyang University College of Medicine, Seoul, Republic of Korea

^2^Biostatistics Collaboration Unit, Yonsei University College of Medicine, Seoul, Republic of Korea

^3^Department of Statistics and Data science, Yonsei University, Seoul, Republic of Korea

^4^Department of Urology, Inha University School of Medicine, Seoul, Republic of Korea

^5^Department of Urology, Ewha Womans University Medical Center, Seoul, Republic of Korea

^6^Department of Urology, Prostate Cancer Center, Gangnam Severance Hospital, Yonsei University College of Medicine, Seoul, Republic of Korea

Supplementary Table 1**.** Diagnostic codes and definitions of comorbidities

| Comorbidity | ICD-10-CM code and definition |
| --- | --- |
| Rheumatoid arthritis | M05 (Seropositive rheumatoid arthritis): M05.0, M05.1, M05.2, M05.3, M05.8, M05.9  M06 (Other rheumatoid arthritis): M06.0, M06.1, M06.2, M06.3, M06.4, M06.8, M06.9 |
| Diabetes mellitus | E10 (Type 1 diabetes mellitus): E10.0, E10.1, E10.2, E10.3, E10.4, E10.5, E10.6, E10.7, E10.8, E10.9  E11 (Type 2 diabetes mellitus): E11.0, E11.1, E11.2, E11.3, E11.4, E11.5, E11.6, E11.7, E11.8, E11.9  E12 (Malnutrition-related diabetes mellitus): E12.0, E12.1, E12.2, E12.3, E12.4, E12.5, E12.6, E12.7, E12.8, E12.9  E13 (Other specified diabetes mellitus): E13.0, E13.1, E13.2, E13.3, E13.4, E13.5, E13.6, E13.7, E13.8, E13.9  E14 (Unspecified diabetes mellitus): E14.0, E14.1, E14.2, E14.3, E14.4, E14.5, E14.6, E14.7, E14.8, E14.9 |
| Hyperthyroidism | E05 (Thyrotoxicosis [hyperthyroidism]): E05.0, E05.1, E05.2, E05.3, E05.4, E05.5, E05.8, E05.9 |
| Chronic liver disease | K73 (Chronic hepatitis): K73.0, K73.1, K73.2, K73.8, K73.9  K74 (Fibrosis and cirrhosis of liver): K74.0, K74.1, K74.2, K74.3, K74.4, K74.5, K74.6  B18 (Chronic viral hepatitis): B18.0, B18.1, B18.2, B18.8, B18.9 |
| Chronic kidney disease | N18 (Chronic kidney disease): N18.1, N18.2, N18.3, N18.4, N18.5, N18.9 |
| COPD | J44 (Other chronic obstructive pulmonary disease): J44.0, J44.1, J44.8, J44.9 |
| Neurological disease |  |
| Stroke | I60 (Subarachnoid haemorrhage): I60.0, I60.1, I60.2, I60.3, I60.4, I60.5, I60.6, I60.7, I60.8, I60.9  I61 (Intracerebral haemorrhage): I61.0, I61.1, I61.2, I61.3, I61.4, I61.5, I61.6, I61.8, I61.9  I62 (Other nontraumatic intracranial haemorrhage): I62.0, I62.1, I62.9  I63 (Cerebral infarction): I63.0, I63.1, I63.2, I63.3, I63.4, I63.5, I63.6, I63.8, I63.9  I64 (Stroke, not specified as haemorrhage or infarction) |
| Parkinson’s disease | G20 (Parkinson’s disease)  G21 (Secondary parkinsonism): G21.1, G21.2, G21.3, G21.4, G21.8, G21.9  G22 (Parkinsonism in diseases classified elsewhere) |
| Dementia | F00 (Dementia in Alzheimer’s disease): F00.0, F00.1, F00.2, F00.9  F01 (Vascular dementia): F01.0, F01.1, F01.2, F01.3, F01.8, F01.9  F02 (Dementia in other diseases classified elsewhere): F02.0, F02.1, F02.2, F02.3, F02.4, F02.8  F03 (Unspecified dementia): F05.0, F05.1 |
| Osteoporosis | M80 (Osteoporosis with pathological fracture): M80.5, M80.8, M80.9  M81 (Osteoporosis without pathological fracture): M81.5, M81.8, M81.9 |
| Fracture |  |
| Hip | S72 (Fracture of femur): S72.0, S72.1, S72.2, S72.3, S72.4, S72.7, S72.8, S72.9 |
| Spine | S22.0 (Fracture of thoracic vertebra)  S22.1 (Multiple fracture of thoracic spine)  S32.0 (Fracture of lumbar vertebra) |
| Upper extremity | S52.5 (Fracture of lower end of radius)  S42.2 (Fracture of upper end of humerus) |

COPD, chronic obstructive pulmonary disease

Supplementary Table 2. Medication code definitions

| Medication | Billing Code |
| --- | --- |
| GnRH agonists or antagonist | Degarelix: 624401BIJ, 624402BIJ  Goserelin acetate: 167202BIJ, 167201BIJ  Leuprorelin acetate: 182602BIJ, 182605BIJ, 182601BIJ, 182604BIJ, 182606BIJ, 182630BIJ, 182608BIJ, 182610BIJ, 182611BIJ  Triptorelin acetate: 244902BIJ, 467501BIJ, 467502BIJ |
| Anti-androgen | Cyproterone acetate: 139401ATB  Bicalutamide: 117201ATB, 117202ATB  Flutamide: 162101ATB |
| Bisphosphonate | Disodium salt of a nitrogen-free bisphosphonate analog: 136101ACH, 136102BIJ, 147401ATB, 207901ACS, 207902BIJ  Alendronate: 228301ATB, 228302ATB, 228303ALQ, 228303ATB  Zoledronic acid: 420702BIJ, 420730BIJ, 420731BIJ  Risedronic acid: 442301ATB, 442302ATB, 442302ATE, 442303ATB, 442330ATB  Ibandronic acid: 480301BIJ, 480302BIJ, 480304ATB, 480330BIJ, 641201BIJ  Combination: 468000ATE, 481100ATB, 500200ATB, 511200ATB, 518400ATB, 523900ATB |
| Calcium, combination with vitamin D and/or other durgs | Calcium carbonate combination: 302600ATB, 303200ATB, 387900ACS, 475200ATB, 480200ATB, 498200ATB, 498300ATB, 526100ATB, 634000ATB  Calcium citrate combination: 302800ATB, 462700ATB, 462800ATB, 503100ATB, 508700ATB, 519000ATB, 665600ATB, 670000ATB |
| Parathyroid hormone | Teriparatide: 646301BIJ, 487502BIJ |

Supplementary Table 3. Age-adjusted Cox regression analysis for osteoporosis in the unmatched (A) and matched (B) cohorts after adjusting for the index date

| **Variables** | **Univariable anlaysis** | |  | **Age-adjusted Cox regression analysis** | | | | | | | |
| --- | --- | --- | --- | --- | --- | --- | --- | --- | --- | --- | --- |
|  |  |  |  | **ADT** | |  | **Duration of ADT** | | | | |
|  | **HR (95% CIs)** | **p-value** |  | **HR (95% CIs)** | **p-value** |  | **HR (95% CIs)** | **pairwise comparison p-value** | | | |
| **(A) Unmatched cohorts** |  |  |  |  |  |  |  |  |  |  |  |
| Age | 1.064 (1.062-1.067) | < 0.0001 |  | 1.059 (1.057-1.062) | < 0.0001 |  | 1.059 (1.056-1.062) | < 0.0001 |  |  |  |
| ADT |  |  |  |  |  |  |  |  |  |  |  |
| No | Ref. |  |  | Ref. |  |  |  |  |  |  |  |
| Yes | 2.069 (1.977-2.165) | < 0.0001 |  | 1.346 (1.283-1.413) | < 0.0001 |  |  |  |  |  |  |
| Duration of ADT |  |  |  |  |  |  |  |  |  |  |  |
| No | Ref. |  |  |  |  |  | Ref. | Ref |  |  |  |
| < 1 year | 1.454 (1.320-1.601) | < 0.0001 |  |  |  |  | 1.013 (0.919-1.117) | 0.795 | Ref |  |  |
| 1~2 year | 1.987 (1.815-2.176) | < 0.0001 |  |  |  |  | 1.270 (1.158-1.393) | < 0.0001 | 0.0005 | Ref |  |
| 2~3 year | 2.048 (1.863-2.250) | < 0.0001 |  |  |  |  | 1.333 (1.211-1.467) | < 0.0001 | < 0.0001 | 0.4534 | Ref |
| > 3 year | 2.476 (2.331-2.630) | < 0.0001 |  |  |  |  | 1.572 (1.477-1.674) | < 0.0001 | < 0.0001 | < 0.0001 | 0.0023 |
| **(B) Matched cohorts** |  |  |  |  |  |  |  |  |  |  |  |
| Age | 1.036 (1.032-1.040) | < 0.0001 |  | 1.035 (1.032-1.039) | < 0.0001 |  | 1.034 (1.031-1.038) | < 0.0001 |  |  |  |
| ADT |  |  |  |  |  |  |  |  |  |  |  |
| No | Ref. |  |  | Ref. |  |  |  |  |  |  |  |
| Yes | 1.363 (1.288-1.442) | < 0.0001 |  | 1.351 (1.277-1.430) | < 0.0001 |  |  |  |  |  |  |
| Duration of ADT |  |  |  |  |  |  |  |  |  |  |  |
| No | Ref. |  |  |  |  |  | Ref. | Ref |  |  |  |
| < 1 year | 0.958 (0.865-1.061) | 0.413 |  |  |  |  | 0.990 (0.893-1.096) | 0.8402 | Ref |  |  |
| 1~2 year | 1.286 (1.167-1.418) | < 0.0001 |  |  |  |  | 1.262 (1.145-1.391) | < 0.0001 | < 0.0001 | Ref |  |
| 2~3 year | 1.336 (1.208-1.477) | < 0.0001 |  |  |  |  | 1.324 (1.197-1.464) | < 0.0001 | < 0.0001 | 0.6389 | Ref |
| > 3 year | 1.652 (1.542-1.771) | < 0.0001 |  |  |  |  | 1.610 (1.502-1.725) | < 0.0001 | < 0.0001 | < 0.0001 | < 0.0001 |

ADT, androgen deprivation therapy; CI, confidence interval; HR, hazard ratio

Supplementary Table 4. Age-adjusted Cox regression analysis for predicting fractures in the unmatched (A) and matched (B) cohorts after adjusting for the index date

| **Variables** | **Univariable analysis** | |  | **Age-adjusted Cox regression analysis** | | | | | | | |
| --- | --- | --- | --- | --- | --- | --- | --- | --- | --- | --- | --- |
|  |  |  |  | **ADT** | |  | **Duration of ADT** | | | | |
|  | **HR (95% CIs)** | **p-value** |  | **HR (95% CIs)** | **p-value** |  | **HR (95% CIs)** | **pairwise comparison p-value** | | | |
| **(A) Unmatched cohorts** |  |  |  |  |  |  |  |  |  |  |  |
| Age | 1.078 (1.075-1.081) | < 0.0001 |  | 1.066 (1.063-1.069) | < 0.0001 |  | 1.066 (1.063-1.069) | < 0.0001 |  |  |  |
| ADT |  |  |  |  |  |  |  |  |  |  |  |
| No | Ref. |  |  | Ref. |  |  |  |  |  |  |  |
| Yes | 2.936 (2.792-3.087) | < 0.0001 |  | 1.843 (1.746-1.944) | < 0.0001 |  |  |  |  |  |  |
| Duration of ADT |  |  |  |  |  |  |  |  |  |  |  |
| No | Ref. |  |  |  |  |  | Ref. | Ref |  |  |  |
| < 1 year | 2.166 (1.959-2.394) | < 0.0001 |  |  |  |  | 1.470 (1.328-1.627) | < 0.0001 | Ref |  |  |
| 1~2 year | 3.034 (2.762-3.334) | < 0.0001 |  |  |  |  | 1.865( 1.694-2.053) | < 0.0001 | 0.0003 | Ref |  |
| 2~3 year | 3.255 (2.958-3.582) | < 0.0001 |  |  |  |  | 2.047 (1.857-2.256) | < 0.0001 | < 0.0001 | 0.1477 | Ref |
| > 3 year | 3.186 (2.981-3.406) | < 0.0001 |  |  |  |  | 1.944 (1.814-2.084) | < 0.0001 | < 0.0001 | 0.4437 | 0.3504 |
| **(B) Matched cohorts** |  |  |  |  |  |  |  |  |  |  |  |
| Age | 1.068 (1.064-1.073) | < 0.0001 |  | 1.068(1.063-1.072) | <.0001 |  | 1.067 (1.063-1.072) | < 0.0001 |  |  |  |
| ADT |  |  |  |  |  |  |  |  |  |  |  |
| No | Ref. |  |  | Ref. |  |  |  |  |  |  |  |
| Yes | 1.800 (1.689-1.918) | < 0.0001 |  | 1.775(1.666-1.892) | <.0001 |  |  |  |  |  |  |
| Duration of ADT |  |  |  |  |  |  |  |  |  |  |  |
| No | Ref. |  |  |  |  |  | Ref. | Ref |  |  |  |
| < 1 year | 1.328 (1.192-1.479) | < 0.0001 |  |  |  |  | 1.415 (1.270-1.577) | < 0.0001 | Ref |  |  |
| 1~2 year | 1.850 (1.669-2.050) | < 0.0001 |  |  |  |  | 1.787 (1.613-1.980) | < 0.0001 | 0.0005 | Ref |  |
| 2~3 year | 1.988 (1.792-2.205) | < 0.0001 |  |  |  |  | 1.962 (1.769-2.177) | < 0.0001 | < 0.0001 | 0.1486 | Ref |
| > 3 year | 1.960 (1.814-2.118) | < 0.0001 |  |  |  |  | 1.874 (1.735-2.025) | < 0.0001 | < 0.0001 | 0.3878 | 0.4064 |

ADT, androgen deprivation therapy; CI, confidence interval; HR, hazard ratio

Supplementary Table 5. Age-adjusted Cox regression analysis for osteoporosis (A) and fracture (B) in the matched cohorts according to the use of anti-androgens

| **Variables** | **Univariable analysis** | |  | **Age-adjusted Cox regression analysis** | |
| --- | --- | --- | --- | --- | --- |
|  | **HR (95% CIs)** | **p-value** |  | **HR (95% CIs)** | **p-value** |
| **(A) Osteoporosis** |  |  |  |  |  |
| Age | 1.031 (1.018-1.045) | < 0.0001 |  | 1.031 (1.018-1.045) | < 0.0001 |
| Anti-androgen use |  |  |  |  |  |
| No | Ref. |  |  | Ref. |  |
| Yes | 1.034 (0.846-1.264) | 0.7436 |  | 1.039 (0.850-1.269) | 0.7120 |
| **(B) Fracture** |  |  |  |  |  |
| Age | 1.075 (1.060-1.091) | < 0.0001 |  | 1.076 (1.060-1.092) | < 0.0001 |
| Anti-androgen use |  |  |  |  |  |
| No | Ref. |  |  | Ref. |  |
| Yes | 1.200 (0.974-1.478) | 0.0874 |  | 1.222 (0.992-1.506) | 0.0599 |

CI, confidence interval; HR, hazard ratio
